# The potential impact of systemic anti-inflammatory therapies in psoriasis on major adverse cardiovascular events: a Korean nationwide cohort study

**DOI:** 10.1038/s41598-021-87766-y

**Published:** 2021-04-21

**Authors:** Joo Ran Hong, Hojin Jeong, Hyeongsu Kim, Hyun Suk Yang, Ji Youn Hong, Sung Min Kim, Young Ah Cho, Yang Won Lee, Yong Beom Choe, Kyu Joong Ahn

**Affiliations:** 1 Department of Dermatology, Konkuk University School of Medicine, 120-1 Neungdong-ro, Gwangjin-gu, Seoul, 143-729 Republic of Korea; 2 Department of Preventive Medicine, Konkuk University School of Medicine, Seoul, Republic of Korea; 3 Department of Cardiovascular Medicine, Konkuk University School of Medicine, Seoul, Republic of Korea; 4 Research Institute of Medical Science, Konkuk University School of Medicine, Seoul, Republic of Korea

**Keywords:** Epidemiology, Cardiovascular diseases

## Abstract

This nationwide population-based cohort study aimed to investigate the impact of systemic anti-inflammatory treatment on the major adverse cardiovascular events (MACE) risk in patients with psoriasis from January 2006 to December 2018, using a database provided by the Korean National Health Insurance Service. Patients were grouped based on the following treatment modalities: biologics, phototherapy, methotrexate, cyclosporine, and mixed conventional systemic agents. Patients who had not received any systemic treatment were assigned to the control cohort. The incidence of MACE per 1000 person-year was 3.5, 9.3, 12.1, 28.4, 39.5, and 14.5 in the biologic, phototherapy, methotrexate, cyclosporine, mixed conventional systemic agents, and control cohorts, respectively. During the 36-month follow-up, the cumulative incidence of MACE in the phototherapy and biologic cohorts remained lower than that of other treatment modalities. Cyclosporine (hazard ratio (HR) = 2.11, 95% confidence interval (CI) = 1.64–2.71) and mixed conventional systemic agents (HR = 2.57, 95% CI = 2.05–3.22) treatments were associated with increased MACE risk. Methotrexate treatment was not associated with MACE. Our finding demonstrates that treatment modalities may affect cardiovascular comorbidities in patients with psoriasis. Thus, an appropriate combination of anti-psoriatic therapies should be considered to manage patients with high cardiovascular risk.

*IRB approval status*: Waiver decision was obtained by the institutional review board, Konkuk University Hospital, Seoul, Republic of Korea (KUH1120107).

## Introduction

Psoriasis is a systemic inflammatory disease that affects approximately 1–3% of the adult population^1^. Epidemiological studies have shown an increased prevalence of cardiovascular (CV) diseases and their metabolic risk factors in patients with psoriasis^2^. Moreover, psoriasis has been suggested as an independent risk factor of major adverse CV events (MACE)^3–6^. The mechanism linking CV disease and psoriasis remains unclear. The pathophysiology of psoriasis is characterized by T-cell-mediated chronic inflammation, which contributes to the pro-atherogenic environment of the disease^7,8^.

In the last few decades, psoriasis treatment has significantly progressed with the identification of multiple new therapeutic targets and the introduction of biological therapies such as tumor necrosis factor-alpha (TNF-α), interleukin (IL)-12/23, and IL-17 inhibitors. Some systemic drugs, specifically biologics, which act by reducing disease-specific inflammation, are expected to improve the CV risk in psoriasis^9^. However, the association between MACE and each anti-inflammatory treatment for psoriasis remains unclear^10,11^. The selection of appropriate treatments for psoriasis has become a complex process considering the coexisting CV comorbidities and the consequent impact of therapeutic agents. Therefore, the association of each therapeutic agent with MACE must be clarified. In line with this, this population-based nationwide cohort study aimed to examine the incidence of MACE in patients with psoriasis and determine the effect of systemic anti-inflammatory treatments on MACE risk.

## Methods

### Data source

This nationwide population-based cohort study was conducted using the customized database of the National Health Insurance Service (NHIS) of Korea (NHIS-2020-1-062). As national insurance in South Korea is mandatory by law, the NHIS database contains data on all health care use of the entire population. The NHIS established the population-based database in 2002, which has registered information on every charged medical and pharmacy claim on all health care uses. Information regarding mortality was obtained from the National Death Registry. Personal information was deidentified and kept protected. The institutional review board of our institution approved a waiver for our study (KUH1120107).

### Patient selection and study design

**Figure 1:**
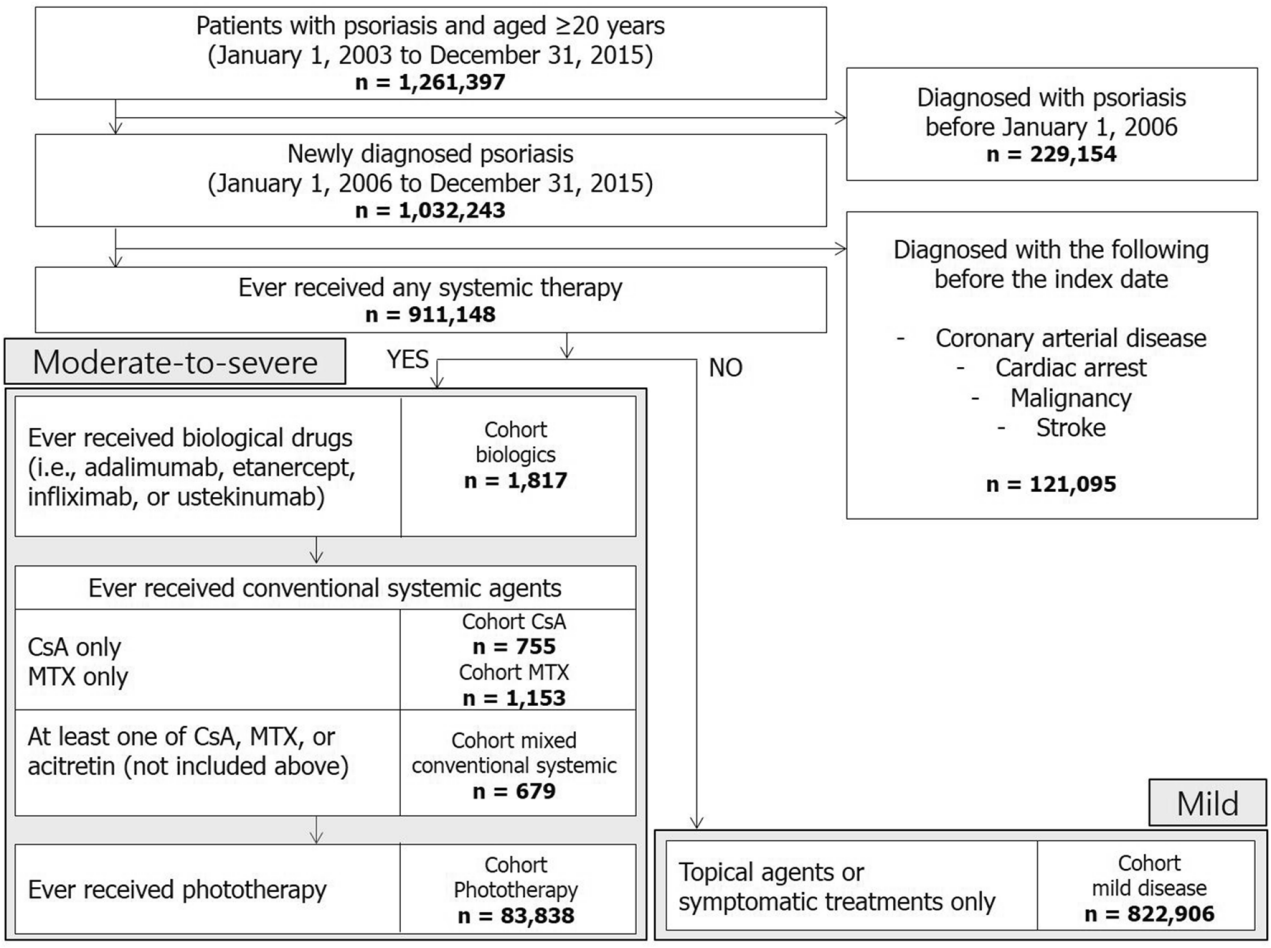
Flowchart of study population selection. CsA: cyclosporine; MTX: methotrexate.

A total of 1,261,397 individuals aged ≥ 20 years who visited clinics or hospitals and had a diagnosis of psoriasis or psoriatic arthritis (*International Classification of Diseases, Tenth Revision* [*ICD-10*] codes: L40.0, L40.1, L40.4, L40.5, L40.8, L40.9, M07.0, M07.2, M07.3, and M09.0) between January 1, 2006, and December 31, 2015, were considered for inclusion in our study. Individuals who were diagnosed with psoriatic disease before 2006 (n = 229,154) were excluded. Furthermore, 121,095 individuals with a history of coronary arterial disease (*ICD-10 codes*: I20–I24), cardiac arrest (I46), stroke (I60–I64), or malignancy (C00-C97) diagnosed before the index date were excluded. Finally, 911,148 patients were enrolled in the study (Fig. 1).

Each patient was assigned to one of six mutually exclusive cohorts. Patients with psoriasis who received at least one dose of etanercept, infliximab, adalimumab, or ustekinumab were assigned to the biologic cohort, regardless of the discontinuation or combined use of other oral agents or phototherapy. Biologic-naive patients who received conventional systemic agents (i.e., cyclosporine, methotrexate, or acitretin) were assigned to the conventional systemic cohort. Among the patients in the conventional systemic cohort, those treated with cyclosporine alone were assigned to the cyclosporine cohort. Patients who received methotrexate exclusively were assigned to the methotrexate cohort, while the remaining patients were assigned to the mixed conventional systemic cohort. Patients who were not treated with biologics or conventional systemic agents but received phototherapy were assigned to the phototherapy cohort. The biologic, cyclosporine, methotrexate, mixed conventional systemic, and phototherapy cohorts were considered to have moderate-to-severe psoriasis, which indicates that the patients had received some systemic treatment. Patients who did not receive any systemic treatment but received only symptomatic or topical treatments were assigned to the mild control cohort.

### Outcomes and follow-up

Data were analyzed from January 1, 2006, to December 31, 2018, with a maximum patient follow-up duration of 3 years. The clinical endpoint of interest was MACE. MACE were defined as the diagnosis of one of the following events recorded in a medical claim for an inpatient stay or outpatient visit: (1) coronary arterial disease (*ICD-10* codes: I20–I24), including angina pectoris (I20) and myocardial infarction (I21–I24); (2) cardiac arrest (I46); (3) stroke (I60–I64), including ischemic stroke (I63) and hemorrhagic stroke (I60–I62); and (4) all-cause mortality. The patients were observed from their index date until the occurrence of their first MACE or until they reached the end of their observation period of 3 years without MACE.

The patients’ index date was defined as the initiation date of systemic treatment for the moderate-to-severe disease groups, and as the date of the first diagnosis of psoriasis or psoriatic arthritis for the mild control group. The baseline period was defined as the 365 days before the index date. The CV risk factors identified during the baseline period included hypertension (*ICD-10* code: I10–I15), dyslipidemia (E78), diabetes mellitus (E10–E14), and end-stage renal disease (N18.5). The *ICD-10* codes used are summarized in Supplementary Table 1.

### Statistical analyses

The Statistical Analysis System (SAS) Enterprise Guide 7.1 software (SAS Institute Inc., Cary, NC, USA; https://www.sas.com) was used for all statistical analyses. Descriptive statistics were used for all variables. *P*-values were calculated using analysis of variance for continuous variables and the *χ*^2^ test for categorical variables. The incidence rates of the study endpoints were calculated as events per 1000 person-years (PYs). Multivariate Cox proportional hazard models were used to compare the MACE hazard among each cohort, adjusting for the potential confounding factors of age, sex, and CV risk factors, with significant differences among the cohorts during the baseline period. The results were reported as hazard ratios (HRs) with their corresponding 95% confidence intervals (CIs) and *P-*values. *P*-values of < 0.05 were considered statistically significant.

## Results

### Demographic characteristics

A total of 911,148 patients with psoriasis were included, of whom 9.7% (n = 88,242) were treated with systemic anti-inflammatory treatments and classified into the moderate-to-severe disease group (Table 1). Among the moderate-to-severe disease group, 2.1% (n = 1817) belonged to the biologic cohort, 2.9% (n = 2587) to the conventional systemic cohort, and the remaining 95% (n = 83,838) to the phototherapy cohort, respectively. The conventional systemic cohort was further divided into three sub-cohorts as follows: the methotrexate cohort included those who were treated with methotrexate only (n = 1153); the cyclosporine cohort included those treated with cyclosporine only (n = 755); and the mixed conventional systemic cohort included those treated with acitretin alone or at least two of the following drugs: methotrexate, cyclosporine, or acitretin (n = 679).

**Table 1:** Study population characteristics stratified according to treatments.

	Moderate-to-severe disease					Mild disease n = 822,906	*P* value^a^
	Biologic n = 1817	Methotrexate n = 1153	Cyclosporine n = 755	Mixed conventional systemic n = 679	Phototherapy n = 83,838		
Male sex, n (%)	1215 (66.9)	654 (56.7)	469 (62.1)	512 (75.4)	43,939 (52.4)	43,2497 (52.6)	** < .001**
Age, y (mean ± SD)	37.1 ± 13.1	43.0 ± 16.2	44.9 ± 16.8	48.6 ± 16.1	40.5 ± 15.0	46.9 ± 16.1	** < .001**
**Range in year, n**							
20–30	670	316	189	103	26,344	152,601	
31–40	458	247	135	117	20,265	164,007	
41–50	390	216	150	152	15,613	172,519	
51–60	214	187	140	129	11,822	156,957	
61–70	61	116	78	118	6,369	101,953	
71-	24	71	63	60	3,425	74,869	
**Baseline comorbidities**							
Hypertension, n (%)	428 (23.6)	284 (24.6)	219 (29.0)	242 (35.6)	14,993 (17.9)	203,745 (24.8)	** < .001**
Diabetes mellitus, n (%)	283 (15.6)	193 (16.7)	184 (24.4)	156 (23.0)	10,029 (12.0)	124,556 (15.1)	** < .001**
Dyslipidemia, n (%)	645 (35.5)	338 (29.3)	236 (31.3)	209 (30.8)	15,195 (18.1)	168,981 (20.5)	** < .001**
End-stage renal disease, n (%)	4 (0.2)	2 (0.2)	21 (2.8)	8 (1.2)	688 (0.8)	2,879 (0.3)	** < .001**
Person-years (mean ± SD)	2.99 ± 0.01	2.98 ± 0.02	2.95 ± 0.06	3.22 ± 0.55	2.98 ± 0.02	2.97 ± 0.03	

^a^Statistically significant at the 5% level.

All cohorts comprised a smaller proportion of women (*P* < 0.001). The mean patient age was 46.3 ± 16.1 years. Patients in the biologic cohort were the youngest among all the groups and were 10 years younger than those in the control cohort (*P* < 0.001).

### Composite cardiovascular endpoint

In total, there were 37,410 patients diagnosed MACE during follow-up. Among them, 21,944 patients were composite CV outcomes, which excludes all-cause mortality from MACE. The individual number and incidence of CV outcomes is shown in Table 2. The incidence rates of MACE were 3.5, 9.3, 12.5, 28.4, and 39.5 per 1000 PYs in the biologic, phototherapy, methotrexate, cyclosporine, and mixed conventional systemic agents cohorts, respectively, compared to 14.5 in the mild control cohort. The mean follow-up duration of patients who developed their first MACE event was 1.4 ± 0.64 years. The incidence of composite CV outcomes was lower for the biologic, phototherapy, methotrexate, and cyclosporine cohorts (2.0, 5.2, 3.8, and 7.6 per 1000 PYs, respectively) and higher for the mixed conventional systemic agents cohort (15.6) than the control cohort (8.4). The highest number of cumulative events for stroke (4.3%) and cardiac arrest (0.3%) may have contributed to the high incidence of CV outcomes in the conventional systemic cohort.

**Table 2:** Incidence rates per 1000 person-years.

Number of events, n (%)		Moderate-to-severe disease					Mild disease n = 822,906
		Biologic n = 1817	Methotrexate n = 1153	Cyclosporine n = 755	Mixed conventional systemic n = 679	Phototherapy n = 83,838	
Composite endpoint including all-cause mortality (MACE)		19 (1.0)	41 (3.6)	61 (8.1)	75 (11.0)	2300 (2.7)	34,914 (4.2)
Mortality^a^		8 (0.4)	28 (2.4)	44 (5.8)	44 (6.5)	997 (1.2)	14,345 (1.7)
Composite endpoint excluding all-cause mortality (Only CV)		11 (0.6)	13 (1.1)	17 (2.3)	31 (4.6)	1303 (1.6)	20,569 (2.5)
Coronary arterial disease		0	0	0	0	44 (0.1)	738 (0.1)
Total stroke^b^		10 (0.6)	13 (1.1)	16 (2.1)	29 (4.3)	1165 (1.4)	18,794 (2.3)
Ischemic stroke		8 (0.4)	8 (0.7)	16 (2.1)	23 (3.4)	894 (1.1)	15,204 (1.8)
Hemorrhagic stroke		1 (0.1)	5 (0.4)	0	6 (0.9)	192 (0.2)	2512 (0.3)
Cardiac arrest		1 (0.1)	0	1 (0.1)	2 (0.3)	94 (0.1)	1037 (0.1)
**Incidence rate per 1000 person-year**							
MACE		3.5	12.1	28.4	39.5	9.3	14.5
Only CV		2.0	3.8	7.6	15.6	5.2	8.4
**Incidence rate per 1000 person-year for age groups**							
Aged ≤ 40	MACE	1.8	1.8	6.2	7.7	1.7	1.7
	Only CV	1.2	0	3.1	3.0	0.8	0.9
Aged 41—60	MACE	4.5	7.6	26.7	30.0	9.8	9.5
	Only CV	2.2	1.7	2.3	12.1	5.9	6.2
Aged ≥ 61	MACE	20.5	56.2	90.8	103.3	46.1	48.8
	Only CV	12.1	20.1	30.2	38.0	25.1	26.8

CV, cardiovascular; MACE, major adverse cardiovascular events.

^a^Classified as “other CV event” when death and CV disease occurred simultaneously on the same day.

^b^Includes strokes not associated with ischemic and hemorrhagic causes.

We further divided the patients into three age groups: ≤ 40, 41–60, ≥ 61 years. The older age group showed a higher cumulative incidence of MACE and composite CV outcomes for all cohorts, excluding the incidence of CV outcome for the cyclosporine cohort aged 41–60 years (2.3 per 1000 PYs). The cyclosporine and mixed conventional systemic cohorts had more than three times higher incidence of composite CV outcome (3.1 and 3.0 per 1000 PYs) than the control cohort aged ≤ 40 years. Meanwhile, the biologic cohort had the lowest incidence of composite CV outcome (12.1 per 1000 PYs) among the study cohorts aged ≥ 61 years.

**Figure 2:**
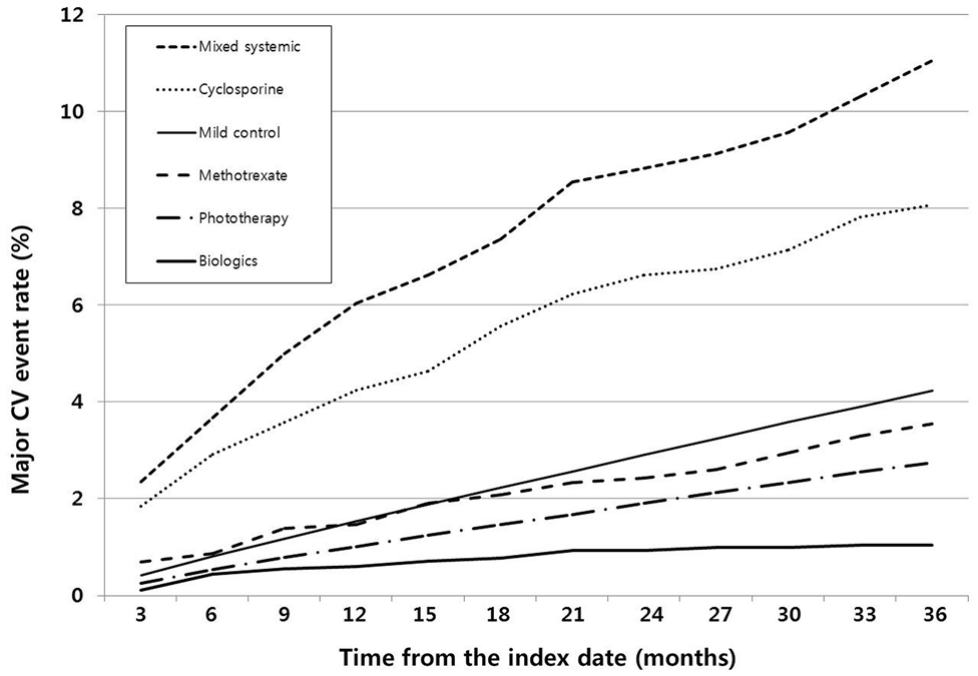
Cumulative incidence of major adverse cardiovascular (CV) events after the initiation of each anti-inflammatory treatment. Cumulative incidence of major CV events every 3 months stratified according to treatment. The difference among each cohort and the control cohort remained statistically significant until the end of the observation period (*P* < .001 at 15 and 30 months), except for the methotrexate cohort (*P* = .98 at 15 months and *P* = .24 at 30 months). CV: cardiovascular.

The stratified cumulative incidence of MACE for each treatment cohort for every 3-month period is shown in Fig. 2. The 36-month cumulative incidence was 1.0, 2.7, 3.6, 8.1, and 11.0% for the biologic, phototherapy, methotrexate, cyclosporine, and mixed conventional systemic agents cohorts, respectively, compared to 4.2% in the mild control cohort. The phototherapy and biologic cohorts showed significantly lower cumulative incidence than the control cohort at any point during the observation periods. Conversely, the cumulative incidence of MACE in the cyclosporine and mixed conventional systemic cohorts remained significantly higher than that in the control cohort until the end of the observation period. However, in this study, the cumulative incidence was not significantly different between the methotrexate and control cohorts.

### Multivariate analysis

Multivariate Cox regression analyses were performed to determine the association of MACE to each treatment modality (Table 3). The phototherapy, biologic, and methotrexate cohorts were not associated with a statistically significant effect regarding MACE when compared with the control cohort. Meanwhile, the cyclosporine (HR = 2.11; 95% CI = 1.64–2.71) and mixed conventional systemic cohorts (HR = 2.57; 95% CI = 2.05–3.22) had a higher MACE risk than the control cohort after adjusting for age, sex, and baseline comorbidities.

**Table 3:** Hazard ratios, and 95% confidence intervals.

	Biologic	Methotrexate	Cyclosporine	Mixed conventional systemic	Phototherapy	Mild disease
HR^a^ (95% CI)	0.87 (0.55–1.36)	1.14 (0.84–1.55)	2.11 (1.64–2.71)	2.57 (2.05–3.22)	1.02 (0.98–1.06)	Reference
*P* value^b^	*P* = .528	*P* = .394	***P < .001***	***P < .001***	*P* = .357	

CI, confidence interval; HR, hazard ratio; MACE, major adverse cardiovascular events.

^a^Adjusted for age, sex, and comorbidities.

^b^Statistically significant at the 5% level.

**Figure 3:**
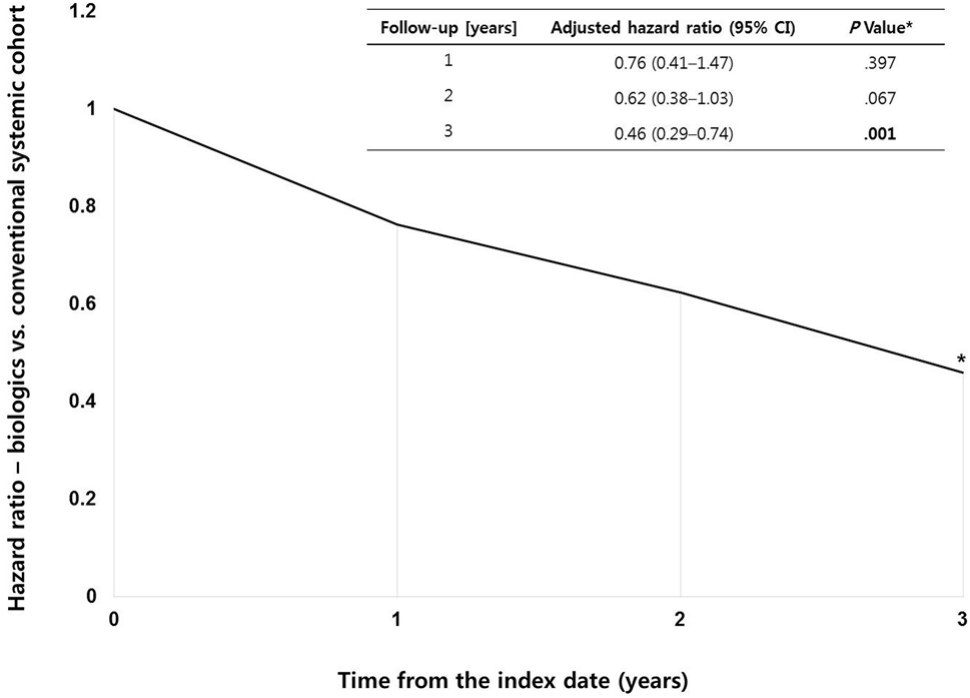
Hazard ratios of the major adverse cardiovascular events between the biologic and conventional systemic treatment cohorts. CI: confidence interval. *Statistical significance at the 5% level.

We further assessed the impact of biologics on the incidence of MACE compared to conventional systemic agents (Fig. 3). The MACE risk associated with biologic treatment tended to decrease over time. Three years after treatment initiation, biologic treatment was associated with a lower MACE risk (HR = 0.46; 95% CI = 0.29–0.74) than treatment with conventional systemic agents.

## Discussion

This cohort study assessed MACE risk according to the treatment modalities of phototherapy, biologic, and conventional systemic agents. Herein, the phototherapy and biologic cohorts showed a lower incidence of MACE than the control cohort, and the difference in the cumulative incidence remained significant during the 36-month follow-up period. The cyclosporine and mixed conventional systemic treatments were significantly associated with increased MACE risk. Methotrexate was not associated with MACE in this study.

The biologic cohort showed the lowest incidence of MACE among all the cohorts. In the present study, the incidence of MACE in the biologic cohort was 3.5 per 1000 PYs. This finding is similar to that of other studies that showed that biologic therapies were associated with lower CV risk than other anti-inflammatory treatments^12–14^. Another study revealed that patients with psoriasis treated with TNF-α inhibitor had a lower risk of a CV event compared to patients treated with phototherapy (adjusted hazard ratio 0.77, *P* < 0.05)^15^. A study conducted in Denmark showed that the incidence was 3.49 per 1000 PYs for a composite CV endpoint in patients with psoriasis treated with TNF-α inhibitors^9^. This is also comparable to the result of a study based on data from the Psoriasis Longitudinal Assessment and Registry, that is, 3.6 per 1000 PYs in patients with psoriasis treated with biologics (n = 7476) or conventional systemic therapies (n = 2019)^16^.

In this study, no coronary arterial disease was recorded in patients treated with biologics. One cardiac arrest case (0.1%) and 10 stroke cases (0.6%) were associated with biologic treatment, and both showed the lowest incidence rates (0.2 and 1.8 per 1000 PYs, respectively). More than half of MACE in the biologic cohort were observed within 1 year (55%), which was higher than that in the control cohort (35%). The difference in the incidence of composite CV outcomes between the biologic and control cohorts was the greatest in the group aged ≥ 60 years (12.1 vs. 26.8 per 1000 PYs), suggesting that the CV protective effect of biologics could be more potent in older individuals. In addition, the most significant MACE risk reductions were observed 3 years after biologic initiation compared to those after conventional systemic treatment (*P* = 0.001). This result implies that biologics that target specific cytokines associated with psoriatic inflammation^17^ may contribute to reducing MACE. However, further investigation with prolonged study periods for individual biological drug classes is required to clarify the association between biologic treatment and MACE risks.

In the present study, the phototherapy treatment was possibly associated with decreased MACE risk. Specifically, the cumulative incidence of MACE was significantly lower with phototherapy than in the control cohort during the study period (*P* < 0.001). In addition, the phototherapy cohort showed a lower incidence rate of acute coronary syndrome and stroke than the control cohort (0.2 and 4.7 per 1000 PYs, respectively). However, based on multivariate Cox regression analyses, phototherapy was not associated with decreased MACE risk compared with the control cohort. Preliminary studies have shown that phototherapy may reduce some inflammatory cytokines; however, there is little evidence regarding a decreased CV risk^18^. Our results suggest that phototherapy may have a positive impact on CV risk in patients with psoriasis.

Methotrexate was not associated with MACE in this study. This result differs from results of previous studies that reported an association between methotrexate treatment and reduced CV risk among patients with inflammatory diseases^19,20^. However, our result may indicate that methotrexate treatment in patients with moderate-to-severe psoriasis may reduce the MACE risk to at least a level comparable to that in patients with mild disease. This is due to the fact that patients with severe psoriasis show higher CV risk than those with mild disease^21^. In addition, the assessment of CV endpoints alone reveals that the methotrexate cohort has the lowest incidence among all the study cohorts in the groups aged ≤ 60 years. Also, coronary arterial disease and cardiac arrest were not related to methotrexate treatment in the present study. Further research is still needed on the effect of methotrexate on CV events.

Treatment with cyclosporine or mixed conventional systemic agents was associated with a significant increase in the incidence of MACE after adjusting for sex, age, and baseline comorbidities. Although the cyclosporine cohort showed a lower incidence of CV outcomes than the control cohort (7.6 *vs*. 8.4 per 1000 PYs), caution is needed to interpret these results. This is because these results may be due to the abrupt decrease in CV events among those in the group aged 41–60 years (2.3 per 1000 PYs), which had a reduced rate of CV events (n = 2) and was consistent with the tendency of increased CV outcomes in older patients in all the other study cohorts.

Specifically, the mixed conventional systemic cohort showed the highest incidence of cardiac arrest and stroke among the cohorts (1.0 and 14.6 per 1000 PYs, respectively). Moreover, the stroke risk was significantly higher in the mixed conventional systemic cohort than in the control cohort after adjusting for age, sex, and comorbidities (HR = 1.60; 95% CI = 1.11–2.31). The cyclosporine cohort had a higher rate of ischemic stroke than the control cohort. For all the age groups, the cyclosporine and mixed conventional systemic cohorts showed a greater than three-fold incidence of CV outcomes in the groups aged ≤ 40 years than in the control cohort. This result may be indicative of the importance of considering CV complications when choosing early systemic anti-inflammatory treatments in younger patients. Previous studies reported that cyclosporine could increase blood pressure^22,23^, total cholesterol levels^24^, and serum creatinine levels^25^. Retinoids, including acitretin, are also associated with increased serum cholesterol and triglyceride levels^26^. This study revealed that caution should be exercised when prescribing cyclosporine or acitretin to patients with high CV comorbidities.

## Limitations

This study has several limitations. First, because patients with certain risk profiles could have been funneled into specific treatment groups, confounding by indication was possible. To overcome this limitation, we considered baseline comorbidities such as hypertension, dyslipidemia, and renal disease as possible variables that could affect the choice of treatment modality. However, the potentially essential baseline comorbidities such as obesity and liver diseases, social factors, treatment dose, and duration of each treatment modality were not evaluated in this study. In addition, we excluded patients who were diagnosed as having the CV outcomes of interest before the index date for clear assessment of the outcomes. This strict removal, whereas, may result in lower incidence in CV outcomes in each cohort than typically expected in patients with psoriasis. Moreover, multivariate Cox regression analyses was not able to done for CV only outcomes due to the small number of outcomes.

Also, age, exposure to each treatment modality, and the baseline characteristics of the patients were analyzed using a fixed model, which limited the accuracy of the allocations of the exposure levels and outcomes in this study. As age is an important confounding factor in CV diseases, we not only included it as a variable for multivariate analysis but also subdivided the patients into three age groups to further exclude the influence of age. The relatively short 3-year follow-up periods also reduced the potential limitation of using age as a fixed variable, although the shorter follow-up period may also constitute a potential limitation of the study.

Additionally, the treatment window, that is, treatment discontinuation after the last claimed treatment, was not considered in this study. This might have been particularly limiting regarding the results in the biologic cohort because the biologics were typically prescribed to patients who had already received phototherapy or conventional systemic agents but had insufficient response to treatment or suffered adverse effects.

Furthermore, clinical data, including laboratory results, are not included in the NIHS database. We were therefore unable to evaluate disease severity or changes in laboratory results.

Finally, the control group was evaluated from the time of diagnosis; thus, theoretically, the duration of the systemic inflammation related to psoriasis was shorter. This might be another limitation of the study, as it caused difficulty in determining whether the increased CV risk was due to the treatment modality or disease severity. However, although our study could not provide accurate interpretation regarding increased CV risk, our results showed that the effectiveness of some systemic treatment modalities, such as cyclosporine or acitretin, for controlling inflammation was not superior to that of other treatment modalities. Furthermore, we could have evaluated whether adequate systemic treatment might lower the CV risk of patients with moderate-to-severe disease compared with those with relatively short disease durations by including patients recently diagnosed with psoriasis.

## Conclusions

This is a population-based nationwide cohort study on the CV risk associated with systemic treatments for psoriasis in Korea. In this study, phototherapy and biologic treatments were associated with decreased incidence rates of MACE. The cyclosporine or mixed conventional systemic agents were associated with an increase in the incidence of MACE; however, methotrexate was not associated with MACE in this study.

Psoriasis and atherosclerosis are chronic inflammatory diseases with similar immune-inflammatory mechanisms. Therefore, patients with psoriasis have high CV morbidity and mortality. Our study showed that adequate anti-inflammatory treatment would decrease the incidence of MACE in patients with moderate-to-severe psoriasis. We should consider an appropriate combination of anti-psoriatic therapies based on each patient’s CV comorbidity to minimize the MACE risk. Randomized trials are required to evaluate the CV safety and efficacy of systemic anti-psoriatic therapies.

## Supplementary Information

Supplementary Information

## Data Availability

All data generated during this study are included in this published article.
