# Supplementary material for: The potential impact of systemic anti-inflammatory therapies in psoriasis on major adverse cardiovascular events: a Korean nationwide cohort study
Source: Sci Rep. 2021 Apr 21;11:8588. doi: 10.1038/s41598-021-87766-y (PMC8060423; doi:10.1038/s41598-021-87766-y)
Supplement: Supplementary file 1 — Supplementary Information [file 41598_2021_87766_MOESM1_ESM.docx]

The potential impact of systemic anti-inflammatory therapies in psoriasis on major adverse cardiovascular events: a Korean nationwide cohort study

Joo Ran Hong, MD, PhD, Hojin Jeong, MS, Hyeongsu Kim, MD, PhD, Hyun Suk Yang, MD, PhD, Ji Youn Hong, MD, Sung Min Kim, MD, Young Ah Cho, MD, Yang Won Lee, MD, PhD, Yong Beom Choe, MD, PhD, and Kyu Joong Ahn, MD, PhD

Supplementary Table 1. International Classification of Diseases (ICD) codes for patients with psoriasis, major cardiovascular outcomes, and baseline comorbidities

| **Disease** | | | **Diagnosis code** |
| --- | --- | --- | --- |
| Psoriasis | | | L40.0, L40.1, L40.4, L40.5, L40.8, L40.9, M07.0, M07.2, M07.3, M09.0 |
| Major adverse cardiovascular event | Coronary arterial disease | |  |
|  |  | Angina pectoris | I20 |
|  |  | Myocardial infarction | I21, I22, I23, I24 |
|  | Cardiac arrest | | I46 |
|  | Stroke |  |  |
|  |  | Ischemic stroke | I63 |
|  |  | Hemorrhagic stroke | I60, I61, I62 |
|  |  | Total stroke | I60, I61, I62, I63, I64 |
| Comorbidities | Hypertension | | I10, I11, I12, I13, I15 |
|  | Diabetes mellitus | | E10, E11, E12, E13, E14 |
|  | Dyslipidemia | | E78 |
|  | End-stage renal disease^a^ | | N18.0, N18.5 |

^a^End-stage renal disease was claimed under N18.0 before January 1, 2010
